# Recent breast cancer incidence trends according to hormone therapy use: the California Teachers Study cohort

**DOI:** 10.1186/bcr2467

**Published:** 2010-01-08

**Authors:** Sarah F Marshall, Christina A Clarke, Dennis Deapen, Katherine Henderson, Joan Largent, Susan L Neuhausen, Peggy Reynolds, Giske Ursin, Pamela L Horn-Ross, Daniel O Stram, Claire Templeman, Leslie Bernstein

**Affiliations:** 1 Department of Epidemiology, School of Medicine, University of California, Irvine, 224 Irvine Hall, Irvine, CA 92697, USA; 2 Northern California Cancer Center, 2201 Walnut Avenue, Suite 300, Fremont, CA 94538, USA; 3 Department of Health Research and Policy and Stanford Cancer Center, Stanford University School of Medicine, 875 Blake Wilbur Drive, Stanford, CA 94305, USA; 4 Department of Preventive Medicine, University of Southern California Keck School of Medicine, Los Angeles, CA 90089, USA; 5 Department of Cancer Etiology, Division of Population Sciences, City of Hope Comprehensive Cancer Center, 1500 East Duarte Road, Duarte, CA 91010, USA; 6 Department of Nutrition, University of Oslo, PO Box 1046, Blindern, 0316 Oslo, Norway; 7 Department of Obstetrics and Gynecology, University of Southern California Keck School of Medicine, Women's and Children's Hospital, 1240 N. Mission Road, Los Angeles, CA 90033, USA

## Abstract

**Introduction:**

Recent, international declines in breast cancer incidence are unprecedented, and the causes remain controversial. Few data sources can address breast cancer incidence trends according to pertinent characteristics like hormone therapy use history.

**Methods:**

We used the prospective California Teachers Study to evaluate changes in self-reported use of menopausal hormone therapy (HT) between 1995 to 1996 and 2005 to 2006 and age-adjusted breast cancer incidence among 74,647 participants aged 50 years or older. Breast cancer occurrence was determined by linkage with the California Cancer Registry.

**Results:**

During 517,286 woman years of follow up, 565 *in situ *and 2,668 invasive breast cancers were diagnosed. *In situ *breast cancer incidence rates in this population did not change significantly from 2000 to 2002 to 2003 to 2005, whereas rates of invasive breast cancer declined significantly by 26.0% from 528.0 (95% confidence intervals (CI) = 491.1, 564.9) per 100,000 women in 2000 to 2002 to 390.6 (95% CI = 355.6, 425.7) in 2003 to 2005. The decline in invasive breast cancer incidence rates was restricted to estrogen receptor-positive tumors. In 1996 to 1999 and 2000 to 2002 invasive breast cancer incidence was higher for women who reported current HT use especially estrogen-progestin (EP) use at baseline than for never or past users; but by 2003 to 2005 rates were comparable between these groups. For women who were taking EP in 2001 to 2002,75% of whom had stopped use by 2005 to 2006, incidence had declined 30.6% by 2003 to 2005 (*P *= 0.001); whereas incidence did not change significantly for those who never took HT (*P *= 0.33).

**Conclusions:**

Few data resources can examine prospectively individual HT use and breast cancer diagnosis. Stable *in situ *breast cancer rates imply consistent levels of screening and suggest recent declines in invasive breast cancer to be explained predominantly by changes in HT use.

## Introduction

Several reports document recent declines in the incidence of invasive breast cancer in the US [1-7] and throughout developed countries [8-13]. The reasons for and timing of these declines is controversial. Most researchers have suggested that the sharp decline observed in 2002 followed widespread reductions in prescribing [14] and use of menopausal hormone therapy (HT) [15], after the July 2002 media coverage of the early termination of the Women's Health Initiative trial of estrogen-progestin (EP) therapy [16]. However, others have argued that changes in mammography use are more likely to be responsible, because subtle declines in breast cancer incidence began in 1999, predating publication of the Women's Health Initiative trial results, and because of the uncertain biological plausibility of an instantaneous change in risk after HT cessation [5]. A sharp decrease in breast cancer incidence in 2002 was reported by at least one cohort defined by regular mammography use [4], but thus far, data documenting incidence trends among women according to their personal HT use are very limited. The ongoing California Teachers Study (CTS) cohort has collected information on participants' HT use periodically since 1995-1996 and is a population with high rates of mammographic screening and HT use [17]. Capitalizing on the capacity to examine long-term trends according to personal HT use history in the CTS, we evaluated changes in breast cancer occurrence over the period from 1996-2005.

## Materials and methods

The CTS, a cohort of female public school teachers and administrators, was established in 1995-1996 to study breast cancer and other women's health issues [17]. The study protocol was approved by the institutional review boards of participating institutions. Participants reported their menopausal status and HT use on the baseline questionnaire. Information on HT use was updated in 2000-2001. This analysis of incidence rates of breast cancer was limited to women who were California residents at baseline and who had no history of *in situ *or invasive breast cancer (n = 118,261). Women who were premenopausal (n = 12,908), had missing data on HT use at baseline or in the 2000-2001 follow-up questionnaire (n = 3222), or did not achieve the age of 50 years (n = 27,484) during the course of the follow-up period (1995-2005) were ineligible for the analyses. As hormones may be taken during the menopausal transition [15], we included women who were either perimenopausal (defined as women who reported that their menstrual periods had stopped within the past six months), postmenopausal (defined as women who reported that their menstrual periods had stopped more than six months ago), or those whose menopausal status was unknown because they were taking HT. As women who were aged 40-49 years at baseline turned 50 years during the follow-up period, they were entered into the analyses. A total of 74,647 women were included in the analyses. HT use was categorized as 'never', 'past', or 'current' in 1995-1996 according to the baseline questionnaire and during follow up in 2000-2001 according to the third questionnaire. For descriptive purposes only, we evaluated how many women had stopped or started HT by the end of follow up in 2005, by examining HT use among 44,108 women who additionally answered HT questions on the 2005-2006 questionnaire. This information is used to illustrate changes in HT use that occurred after 2000-2001; however, the small number of breast cancers that occurred in the interval between 2000-2001 and 2005-2006 precluded subgroup analyses. Informed consent of participants was implied by questionnaire completion.

Incident diagnoses of invasive and *in situ *breast cancer (*International Classification of Diseases *for Oncology, Third Edition (*ICD*-O-3) site codes 500-509, excluding morphology codes 9590-9989) were identified by annual linkage with the California Cancer Registry [18]. Cancer data were complete through to 31 December, 2005. The cohort was followed from the date of completion of the baseline questionnaire until the earliest of the following events: the date of first *in situ *or invasive breast cancer diagnosis, date of death, date of move outside of California, or 31 December, 2005. Continued California residence was self reported on three follow-up questionnaires and was supplemented by the information obtained from the US Postal Service National Change of Address database and change of address cards provided in the annual newsletter that were submitted by participants. Deaths were identified through annual probabilistic record linkage to the California state mortality file, the national Social Security Administration death master file and the National Death Index. Women who moved out of state or died during a calendar year contributed woman months of follow up until the month of move or death. Woman years, as the denominators for incidence rates for each period of analysis, were accrued based on the above described eligibility criteria and are shown in Table 1. We used SAS version 9.2 (SAS Institute, Cary, NC, USA) to calculate age-adjusted incidence rates per 100,000 woman years, standardized to the 2000 US population (10 age groups - Census P25-1130), and corresponding 95% confidence intervals (CI) for three distinct time periods; 1996-1999, 2000-2002, and 2003-2005, a) for invasive breast cancer overall, and by estrogen receptor (ER) status and for *in situ *breast cancer, b) according to HT use reported at baseline, and c) according to HT use reported in 2000-2001. We did not have adequate numbers of cases to conduct regression-based assessments of trends, thus we calculated 95% CI and tested whether the changes between time period-specific rates were statistically significant, treating 2000-2002 as the reference group, using a z score [19]. Where multiple time periods were assessed the *P *values were adjusted for multiple comparisons using a Bonferroni correction [20].

**Table 1:** Age-standardized baseline characteristics of menopausal women in the California Teachers Study according to hormone therapy (HT) use in 1995-1996

	Never HT (n = 14,070)	Past HT (n = 7914)	Current E (n = 15,407)	Current EP (n = 16,934)
Mean age in years	66.5	68.2	63.3	58.9
% Non-Hispanic white	85.3	87.9	90.1	92.2
% First-degree family history of breast cancer	15.2	14.9	13.2	12.2
% BMI overweight or obese ≥25 kg/m^2^	45.2	45.2	41.6	34.9
% High alcohol intake (average ≥20 g/day)	8.6	9.3	9.5	10.4
% Highest quartile of residential socioeconomic status	44.4	44.8	47.5	52.5
% Menarche at age 11 years or younger	22.0	23.4	23.6	20.9
% Nulliparous	23.5	19.5	18.8	19.5
% Parous before age 35 years	70.2	74.8	76.3	75.1

BMI = body mass index; E = estrogen only; EP = estrogen and progestin combined therapy.

Age-standardized by 10-year age categories to the age distribution of menopausal women in analysis

All analyses were repeated to determine the influence of including a small number of women who reported on the baseline questionnaire that they did not receive regular screening for breast cancer. Observed trends were essentially unchanged whether including or excluding these unscreened women. Therefore, we present below results including all women regardless of the regularity of their mammographic screening.

## Results

During 517,286 woman years of follow up, 566 *in situ *and 2668 invasive breast cancers were diagnosed. Among the invasive tumors, 1945 (72.9%) were ER positive; 324 (12.1%) were ER negative; and 399 (15.0%) were borderline or ER status was unknown (Table 1). At baseline, 26.5% of age-eligible participants had never taken HT, 59.9% were current users and 13.5% were past users. In 2000-2001, with an additional 5599 women having aged into the analysis cohort, 23.0% were classified as never HT users, 57.7% were current users and 19.3% were past users. By 2005-2006, 26.9% had never taken HT, 21.0% reported current use and 52.1% were past users. By the end of follow up in 2005-2006, 75.0% of those reporting current EP use in 2000-2001, and 55.8% of those reporting current estrogen-only HT use, had stopped HT use.

### Baseline characteristics according to HT use history

Compared with women who had never taken HT, current users were more likely to be younger and non-Hispanic white (Table 1). Current EP users were more likely than estrogen-only HT users, or past or never HT users to live in an area with the highest socioeconomic status and were much less likely to be overweight or obese. Women who had never taken HT were less likely to have had their first full-term pregnancy before age 35 years. Family history of breast cancer, age at menarche and alcohol intake did not differ by HT use history.

### Breast cancer incidence rates

**Figure 1:**
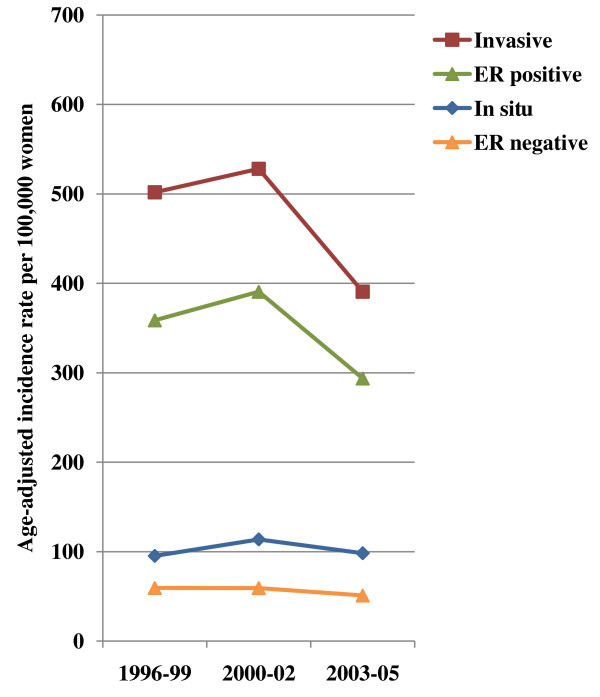
**Age-adjusted incidence rates of breast cancer according to type of tumor and estrogen receptor (ER) status of invasive cancers among menopausal women 50 years or older in the California Teachers Study**.

Overall, age-adjusted incidence rates of *in situ *breast cancer did not change substantially (change from 1996-1999 to 2000-2002, *P *= 0.09; from 2000-2002 to 2003-2005, *P *= 0.24; Figure 1). However, incidence rates of invasive breast cancer peaked in 2000-2002, and then declined by 26.0% from 528.0 (95% CI = 491.1 to 564.9) per 100,000 women in 2000-2002 to 390.6 (95% CI = 355.6 to 425.7) per 100,000 woman years in 2003-2005 (Table 2). The decline in rates from 2000-2002 to 2003-2005 was statistically significant (*P *< 0.001). Examining trends separately by ER status, declines were limited to ER-positive cancer, for which rates peaked in 2000-2002 at 390.6 per 100,000 woman years before declining 24.8% to 293.8 in 2003-2005 (*P *< 0.001). Rates of ER-negative cancer were generally stable throughout the time period; the slight 14.1% decline in rates between 2000-2002 and 2003-2005 was not statistically significant (*P *= 0.38). Among women with invasive breast cancer, 77.8% with ER-positive disease and 70.4% with ER-negative disease reported having used HT at some time on their baseline questionnaire.

**Table 2:** Age-adjusted incidence rate (AAIR) of breast cancer, according to type of tumor and estrogen receptor (ER) status of invasive cancers among menopausal women in the California Teachers Study

Period	Woman	*In situ*		Invasive		ER positive		ER negative	
		
	years	n	AAIR (95% CI)	n	AAIR (95% CI)	n	AAIR (95% CI)	n	AAIR (95% CI)
1996-99	208,860	207	95.2 (81.6-108.7)	1072	501.7 (470.3-533.1)	765	358.7 (332.1-385.3)	127	59.5 (48.6-70.3)
2000-02	158,917	197	113.7 (96.5-130.9)	914	528.0 (491.1-564.9)	676	390.6 (358.8-422.3)	103	59.4 (46.9-71.9)
2003-05	149,509	161	98.2 (79.1-117.3)	682	390.6 (355.6-425.7)	504	293.8 (261.9-325.7)	94	51.0 (40.5-61.5)
Total cases		565		2668		1945		324	

% change 1996-99 to 2000-02			19.4% *P *= 0.09		5.2% *P *= 0.30		8.9% *P *= 0.13		-0.2% *P *= 0.99
% change 2000-02 to 2003-05			-13.6% *P *= 0.24		-26.0% *P *< 0.001		-24.8% *P *< 0.001		-14.1% *P *= 0.38

CI = confidence interval.

### Incidence rates by HT use history

**Figure 2:**
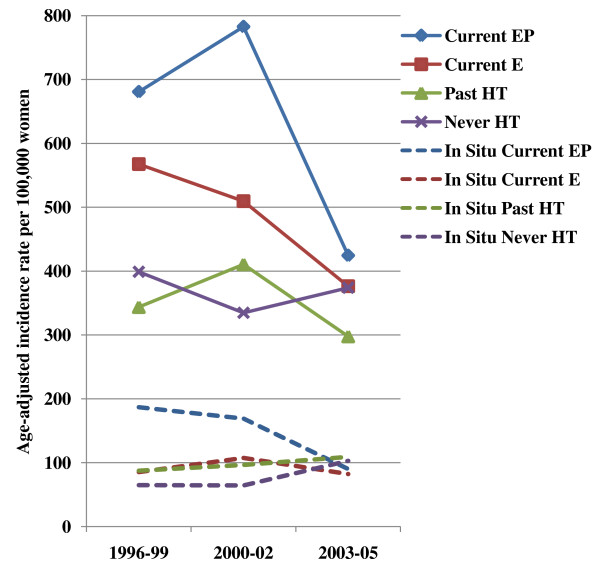
**Age-adjusted incidence rates of invasive (solid lines) and *in situ *(dashed lines) breast cancer according to hormone therapy use at baseline in 1995-1996 among menopausal women 50 years or older in the California Teachers Study**. E = estrogen only hormone therapy; EP = estrogen-progestin hormone therapy; HT = Hormone therapy.

Figure 2 shows invasive and *in situ *breast cancer incidence trends according to HT use reported on the baseline questionnaire. Women who reported currently taking EP at baseline had consistently higher rates of invasive and *in situ *breast cancer than estrogen-only HT users or never or past HT users. Whereas the age-adjusted incidence rates of invasive breast cancer between 2000-2002 and 2003-2005 were stable for never users and past users of HT, among women who reported current use of EP at baseline, incidence rates declined significantly by 45.8% from 782.7 (95% CI = 687.1 to 878.2) per 100,000 in 2000-2002 to 424.6 (95% CI = 353.5 to 495.7) in 2003-2005 (*P *< 0.001; Table 3). Women currently taking EP also experienced the largest decline in rates of *in situ *cancer (percent change = -46.7, *P *= 0.008). Declines in breast cancer rates among current E users were also observed (invasive percent change = -26.1%, *P *= 0.010; *in situ *percent change = -23.3%, *P *= 0.23).

**Table 3:** Age-adjusted incidence rate (AAIR) of invasive and *in situ *breast cancer according to hormone therapy (HT) use at baseline in 1995-1996 among menopausal women in the California Teachers Study

Period	Never HT	Past HT	Current E	Current EP
	
	AAIR (95% CI)	AAIR (95% CI)	AAIR (95% CI)	AAIR (95% CI)
	Invasive breast cancer			

1996-99	399.0 (341.7-456.3)	343.7 (268.8-418.6)	567.5 (501.1-633.9)	680.8 (576.7-784.8)
2000-02	334.7 (278.5-390.9)	410.6 (300.7-520.6)	509.6 (438.9-580.2)	782.7 (687.1-878.2)
2003-05	374.1 (308.1-440.1)	297.8 (218.5-377.2)	376.4 (314.7-438.1)	424.6 (353.5-495.7)

% change 1996-99 to 2000-02	-16.1% *P *= 0.13	19.5% *P *= 0.31	-10.2% *P *= 0.11	15.0% *P *= 0.016
% change 2000-02 to 2003-05	11.8% *P *= 0.38	-27.5% *P *= 0.17	-26.1% *P *= 0.010	-45.8% *P *< 0.001

	*In Situ *breast cancer			

1996-99	65.0 (41.1-88.9)	87.5 (43.2-131.7)	85.4 (60.2-110.6)	187.0 (128.3-245.6)
2000-02	64.5 (39.5-89.6)	96.6 (29.7-163.5)	107.5 (78.1-136.9)	169.2 (128.6-209.9)
2003-05	103.1 (60.5-145.7)	131.7 (163.5-160.6)	82.4 (56.5-108.3)	90.1 (65.4-114.8)

% change 1996-99 to 2000-02	-0.8% *P *= 0.98	10.4% *P *= 0.82	25.9% *P *= 0.12	-9.5% *P *= 0.38
% change 2000-02 to 2003-05	59.8% *P *= 0.10	36.3% *P *= 0.51	-23.3% *P *= 0.23	-46.7% *P *= 0.008

CI = confidence interval; E = estrogen only; EP = estrogen and progestin combined therapy.

**Figure 3:**
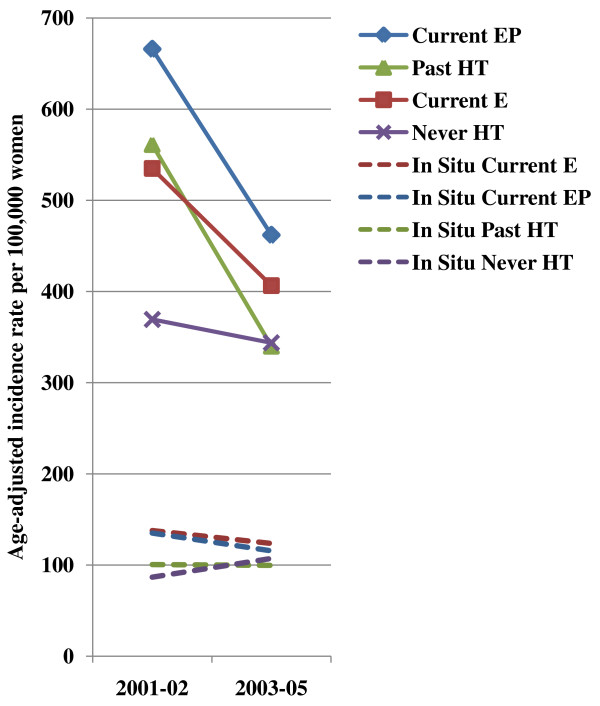
**Age-adjusted incidence rates of invasive (solid lines) and *in situ *(dashed lines) breast cancer according to hormone therapy use at follow-up in 2000-2001 among menopausal women 50 years or older in the California Teachers Study**. E = estrogen only hormone therapy; EP = estrogen-progestin hormone therapy; HT = Hormone therapy.

HT use was reassessed on a second questionnaire administered in 2000. Breast cancer incidence was calculated for two time periods 2001-2002 and 2003-2005 classified according to participants' HT use reported for 2000-2001. Figure 3 and Table 4 show that during this time, invasive and *in situ *breast cancer rates declined for all categories of HT users, except for women who had never taken HT, in whom there was a non-significant increase of 23.8% in *in situ *breast cancer between the two time periods. Age-adjusted incidence rates for invasive breast cancer declined rapidly for women who had stopped HT by 2000-2001 ('past HT use') from 561.1 (95% CI = 432.4 to 689.8) per 100,000 women in 2001-2002 to 340.0 (95% CI = 265.6 to 414.4) per 100,000 in 2003-2005, an overall decline of 39.4% (*P *= 0.003). A similar decline was observed for women who were current users of EP in 2000-2001 (percent change = -30.6, *P *= 0.001), and to a lesser extent, for current estrogen-only HT users (percent change = -24.0, *P *= 0.030). For never users of HT, invasive incidence trends were stable between the two time periods 2001-2002 (percent change = -6.9, *P *= 0.33). None of the changes in rates observed for *in situ *breast cancer were statistically significant.

**Table 4:** Age-adjusted incidence rate (AAIR) of invasive and *in situ *breast cancer according to hormone therapy (HT) use at follow-up in 2000-2001 among menopausal women in the California Teachers Study

Period	Never HT	Past HT	Current E	Current EP
	
	AAIR (95% CI)	AAIR (95% CI)	AAIR (95% CI)	AAIR (95% CI)
	Invasive breast cancer			
2001-02	369.3 (285.6-452.9)	561.1 (432.4-689.8)	534.9 (424.7-645.1)	665.8 (565.9-765.7)
2003-05	343.7 (272.8-414.5)	340.0 (265.6-414.4)	406.4 (324.7-488.0)	461.9 (392.5-531.4)

% change	-6.9% *P *= 0.33	-39.4% *P *= 0.003	-24.0% *P *= 0.030	-30.6% *P *= 0.001

	*In Situ *breast cancer			
2001-02	86.6 (44.0-129.1)	100.5 (32.4-168.6)	137.7 (83.2-192.3)	135.0 (95.2-174.9)
2003-05	107.2 (63.1-151.3)	99.5 (60.3-138.8)	123.6 (67.3-179.9)	115.5 (78.1-152.8)

% change	23.8% *P *= 0.26	-1.0% *P *= 0.49	-10.2% *P *= 0.35	-14.4% *P *= 0.22

CI = confidence interval; E = estrogen only; EP = estrogen and progestin combined therapy.

## Discussion

Researchers using ecologic study designs have only been able to speculate that HT is responsible for the unprecedented decline in invasive breast cancer incidence observed in 2002 [1,2,14,21]. With the individual-level information on HT use history in our prospective cohort, we are able to show several important phenomena regarding breast cancer occurrence in this population: 1) 24.8% decline in ER-positive breast cancer occurring between 2000-2002 and 2003-2005 while ER-negative breast cancer remained stable; 2) incidence trends that varied substantially by HT use at baseline, with consistently higher rates among current HT users particularly EP users, than never users; 3) 45.8% decline in breast cancer incidence from 2000-2002 to 2003-2005 observed among women who were current EP users in 2000-2002, the majority of whom (75.0%) were likely to have ceased use by 2005, but not among women who had never used HT; and 4) stable rates among women who reported never having any HT use. Thus, these data provide further support for the stronger etiologic importance of current versus past HT use in breast cancer risk.

Among women reporting that they stopped using HT, rates of invasive breast cancer rapidly declined. This is demonstrated by both the trends in incidence among women who had stopped use by 2000-2001 ('past users') who had a 39.4% drop in rates and the trends in incidence among women who were still taking EP in 2000-2001, but who had largely stopped use by 2005-2006. Taken together, our observations demonstrate a strong and immediate influence of HT use and cessation on breast cancer occurrence [21].

The CTS population reports near uniformity with respect to screening compliance. Nearly 97% of CTS participants aged over 50 years at baseline reported being current with mammography guidelines at baseline. Stable *in situ *breast cancer rates and stable invasive breast cancer rates among women who had never taken HT imply consistent levels of screening throughout the study period and screening rates are particularly high for this population [17]. Furthermore, when we restricted our analyses to those 97% that were current with breast health screening at the start of our study, the results did not change, and similar declines in breast cancer incidence have been reported from populations of women screened uniformly [4]. Other researchers have even suggested increased usage of mammography [22]. Clearly, any possible influence of 'mammography saturation' or other screening effects will be smaller and more gradual than the recent rapid declines that we suggest are attributable to the dramatic changes in HT use.

We observed large declines in breast cancer incidence; up to 45.8% among current EP users. We feel that this decline can be attributed mostly or entirely to HT use for several reasons: a) this cohort reported high levels of HT use at baseline - more than 70% of menopausal women reported ever taking HT; b) this cohort experienced a large decline in HT use - 75% of the EP users had stopped taken it by 2005 and 56% of the estrogen-only HT alone users had also stopped; c) some studies report more than 66% increased risk due to HT use [23]; and d) the largest declines we observed were in groups that are plausible biologically - that is, current or recent EP users, and in ER-positive tumors. In fact the size and speed of the declines we observed make it unlikely that changes in other risk factors including reproductive history, body mass index, diet or alcohol use are attributable. Some authors [24] have postulated that the increased use of other medications such as raloxifene and tamoxifen may be partly responsible for reducing breast cancer incidence but prevalence of use in the CTS in 2005-2006 was very low (about 8.2%) compared with HT.

Although our data come from one of the largest prospective cohorts tracking HT use, we still had limited statistical power to detect changes in annual breast cancer incidence, especially for disease subgroups. Like prior population-based studies, we also found that decreases in breast cancer incidence were only observed for ER-positive cancers [2-4]; however, we did not have a sufficient number of cases to examine trends of ER-defined breast cancers according to HT use, nor could we examine duration of HT use.

## Conclusions

Along with other studies reaching similar conclusions [1,2,25], our data provide further evidence that recent declines in invasive breast cancer incidence in the US are explained predominantly by decreased HT use.

## Abbreviations

CI: confidence intervals; CTS: California Teachers Study; ER: estrogen receptor; EP: estrogen-progestin hormone therapy; HT: Hormone therapy.

## Competing interests

CAC has served as an expert witness for plaintiff lawyers preparing hormone therapy litigation. All other authors declare that they have no competing interests.

## Authors' contributions

SFM carried out the statistical analysis and drafted the manuscript. CAC and LB conceived and supervised the analysis, helped to draft the manuscript, and participated in the design and coordination of the study. DD, KDH, JL, SLN, PR, GU, PLH-R, DOS, and CT participated in the design of the analysis and edited the manuscript. All authors read and approved the final manuscript.
